# Lynch syndrome related endometrial cancer: clinical significance beyond the endometrium

**DOI:** 10.1186/1756-8722-6-22

**Published:** 2013-03-25

**Authors:** Yiying Wang, Yue Wang, Jie Li, Janiel Cragun, Kenneth Hatch, Setsuko K Chambers, Wenxin Zheng

**Affiliations:** 1 Department of Obstetrics and Gynecology, Henan Province People’s Hospital Zhengzhou, Henan, China; 2 Department of Obstetrics and Gynecology, University of Arizona, Tucson, AZ, USA; 3 Department of Pathology, University of Arizona College of Medicine, Tucson, AZ, USA; 4 Department of Obstetrics and Gynecology, Qilu Hospital, Shandong University, Shandong, China; 5 Arizona Cancer Center, University of Arizona, Tucson, AZ, USA

## Abstract

Lynch syndrome (LS), an autosomal dominant inherited cancer susceptibility syndrome, also known as hereditary non-polyposis colon cancer (HNPCC), is caused by a germline mutation in one of several DNA mismatch repair (MMR) genes. LS is the most common presentation of hereditary colorectal cancer (CRC), accounting for about 2–5% of all CRC cases. More recently, it is found that a similar number of endometrial cancers is also due to one of the MMR gene mutations. There has been significant progress in LS-related CRC in terms of molecular pathogenesis, risks, genetic basis, and cancer prevention. In contrast, the advance about LS-related endometrial cancer (EC) is very much limited. In this commentary, we summarize the main clinicopathologic features of LS-related EC and propose universal screening for LS in individuals with endometrial cancer.

## 

Lynch syndrome (LS), an autosomal dominant inherited cancer susceptibility syndrome, also known as hereditary non-polyposis colon cancer (HNPCC), is caused by a germline mutation in one of several DNA mismatch repair (MMR) genes: MLH1, MSH2, MSH6 or PMS2. LS is the most common presentation of hereditary colorectal cancer (CRC), accounting for about 2–5% of all CRC cases [1,2]. There has been significant progress in LS-related CRC in terms of molecular pathogenesis, risks, genetic basis, and cancer prevention. Moreover, universal screening for LS in all individuals affected by CRC has been adopted by national working groups, with algorithms for cost-effective screening developed [3].

Recently, focus has shifted to LS-related endometrial cancer (EC) as women with LS have a 40-60% chance of presenting with EC as the first clinical manifestation [4]. From a clinical perspective, confirmation that an EC is LS-related has the potential to influence early detection, screening, and prevention of other LS-associated cancers, more so than a diagnosis of LS related CRC. Many countries, including United States, have started screening patients with EC to identify those with LS, thereby leading to earlier screening for CRC. Earlier screening would aim to either prevent CRC or detect it in earlier stages [5]. In that 50% of LS patients present with endometrial cancer first, diagnosis of LS at the time of diagnosis of the endometrial cancer, may prove to be a cost-effective approach, although currently untested. It may be more cost effective mainly because the mortality of CRC is much higher than that of EC. At this time, although several groups nationally are now focusing on LS-related ECs, research on LS-related EC is still evolving. The majority of physicians and health care providers are not aware of the clinicopathologic features of LS-related EC. Further, many are unclear as to how to make the diagnosis of LS-related EC from clinicopathologic perspective and how to best confirm the diagnosis of LS on a genetic level. In this commentary, we summarize the main clinicopathologic features of LS-related EC and propose universal screening for LS in individuals with endometrial cancer.

### The genetic changes in LS-related EC and its clinical impact

Among LS-related EC, the mutation rate of MMR genes shows a frequency in the MSH2 gene of 50-66%, in MLH1 of 24-40%, and in MSH6 of 10-13%, respectively [6]. Compared with EC, the gene mutation rate in LS-related CRC is either similar or slightly less frequent, ie. MSH2 mutation rate is the highest, while the MLH1 mutation is the second most frequent in LS-related EC as well as in LS-related CRCs. Although the overall mutation rate of MSH6 is relatively low (10-13%), it is closely associated with LS-related ECs [6,7], while the rate of PMS2 mutation is less than 5% [8], which has the lowest frequency.

The main function of MMR is to maintain genomic stability by correcting mismatches generated during DNA replication. MMR malfunction results in a mutated phenotype and microsatellite instability (MSI), which promotes cancer formation [9]. In endometrial cancers, MSI is very common and while a hallmark of LS, MSI is not equivalent to LS. MSI is also present in 15–25% of corresponding sporadic cancers [10]. In this context, there are two conditions which may result in MSI. One is where hypermethylation of the MLH1 gene promoter results in gene silencing, leading to MSI. Hypermethylation is an epigenetic event thus is common in patients with sporadic EC. The second condition is where a germ line mutation of one or more of the above mentioned MMR genes can induce MSI resulting in development of LS [11].

Based on the above characteristics, in endometrial cancers, MSI detection is not specific thus not very helpful in the diagnosis of LS. Therefore, a practical approach is needed to find LS-related EC patients for both early detection and prevention of other LS-associated cancers.

### The clinicopathological features of LS-related EC

It is being recognized that, in LS, the risk of EC is equal to, or higher than the risk of CRC. Lifetime risk of LS-related EC is associated with age and a mutation of a specific MMR gene. Patients with *MSH6* mutations are at higher risk (64–71%) for developing EC than those with *MSH2* or *MLH1* mutations (40–50%) [12,13]. They are also at significantly higher risk of developing EC than CRC. It has long been thought that LS-related EC occurs at younger ages than in sporadic cases. In one study, the mean age at diagnosis for LS-related EC was 49 years compared to 60 years for EC in the general population [14]. In an unselected cohort of endometrial cancers with age younger than 50 years, the incidence of LS was 9% [15]. However, others [16] have found the mean age in a prospective unselected cohort to be 54 years. If an age cut-off of 50 years old had been selected instead for LS screening, 60% of patients would have been missed [17]. In fact, others have described that 25% of LS patients do not fit standard screening criteria, such as the Amsterdam, Bethesda, and SGO criteria, where age is a prominent factor (cut-off age 50) [18].

It is interesting to note that LS-related EC sometimes is accompanied (synchronous, or metachronous) by LS-related ovarian cancer. In fact in LS, the lifetime risk for endometrial cancer is 40-60%, and for ovarian cancer, 9-12% [19]. The latter is usually clear cell carcinoma and can present independently and less frequency [4,20]. Compared to LS-related EC cases, LS-related ovarian cancer tends to have a higher *MSH2* mutation rate and occurs in a younger age, average of 45 years old [21]. For patients with LS-related EC, the risk of developing a second cancer is estimated at 25% in 10 years and 50% at 15 years following initial EC diagnosis [22,23]. In LS, 50% of the time, endometrial cancers present first before the CRC diagnosis, if the diagnoses are not synchronous. Therefore, EC can serve as a ‘sentinel’ cancer for patients themselves and potentially for their family members. Optimally, this provides adequate time to screen for a second cancer leading to either prevention or earlier diagnosis and treatment.

Clinically, studies have revealed that patients with LS-related EC have unique features. Both Type 1 and Type 2 ECs are part of LS [24-26]. In the general population, non-endometrioid EC is typically diagnosed in older women with a mean age of 65 to 68 years [27,28]. In LS, however, the mean age of diagnosis of these non-endometrioid tumors is 46.4 years, similar to the mean age of LS-related EC overall (46.8 years) [25]. Patients with LS-related EC often have no evidence of estrogen overstimulation such as obesity, diabetes, exogenous estrogen usage, and polycystic ovarian syndrome. Patients may present irregular bleeding, but it is less likely to be found to have endometrial hyperplasia prior to EC diagnosis. LS-related EC is suspected when a patient is diagnosed with EC, but presents without risk factors known to be associated with EC. In addition, low body mass index, relatively young age, and positive family history LS or LS-related cancers should raise suspicion of LS-related EC [29,30].

Pathologically, there is considerable literature on the presence or absence of distinctive microscopic features in LS-related EC. Historically, many studies correlated the morphologic features of MSI and MLH1 methylation status. In addition, there are many studies correlation LS-related EC to MMR gene mutation status. We summarize the overall pathologic features for LS-related EC as follows:

(1) LS-related ECs tend to be more histologically diverse and can include endometrioid and non-endometrioid histotypes. Clear cell carcinoma, endometrial serous carcinoma, undifferentiated carcinoma, and carcinosarcoma all have been identified as non-endometrioid histology in LS-related ECs [24-26]. In contrast, sporadic ECs that have MSI due to MLH1 methylation are predominantly endometrioid, especially FIGO grades 2 and 3, with the percentage endometrioid histotype reaching 96% [25].

(2) Microscopic features that have been associated with the presence of high level of MSI include poor differentiation, mucinous features, signet ring cell differentiation, mixed tumor histology, tumor cells growing in a medullary-type pattern, increased tumor-infiltrating lymphocytes, and a Crohn-like inflammatory infiltrate at the tumor invading front or periphery [17].

(3) LS-related EC cases also have a tendency to involve low uterine segment (LUS). Westin et al. showed that a relatively high percentage (34%) of the LUS cancers had a high level of MSI. Among them, 29% of the LUS ECs were confirmed to be from women with LS [31]. This percentage of LS-related EC with LUS location is extremely high when compared with the incidence of LUS involvement in non LS-related EC [25,32,33]. In addition, LS-related EC with LUS involvement is also more associated with *hMSH2* mutations [6].

Although the published data for LS-related EC is limited, it is our opinion that these pathologic features are not sufficiently sensitive and specific to be used in the clinical setting as accurate predictors of the presence of LS. However, they do raise suspicion of LS. Therefore, appropriate tissue testing (described below) followed as appropriate with genetics counseling with germline DNA mutational analysis for suspected MMR genes are needed to confirm if LS is present.

### Tissue testing for Lynch syndrome from women with endometrial cancer

Tissue testing (immunohistochemistry for MMR proteins, MSI analysis, and MLH1 methylation analysis) has been used as a practical first step in the evaluation of individuals thought to be at risk for having LS. Practically, each of these tests can be performed using formalin-fixed, paraffin-embedded tissues and commercially available antibodies. In many institutions, MSI analysis is performed in parallel with immunohistochemistry for MLH1, MSH2, MSH6, and PMS2. It requires both tumor and normal non-tumor tissues. For endometrial cancer, adjacent benign endometrium and stromal cells in addition to normal cervix or ovarian tissues could be used as normal controls. For those tumors with MSI and loss of MLH1 by immunohistochemistry, a PCR-based assay to detect for hypermethylation of the MLH1 promoter is performed. If methylation is present, it is much more likely that the patient has a sporadic endometrial carcinoma rather than a LS-related EC. For CRC, BRAF mutation is also an explanation for loss of MLH1 protein expression. However, studies [16] of EC specimens show that BRAF mutation is very rare, thus BRAF mutation testing is not recommended in endometrial cancers when MLH1 loss is seen. The MLH1 and PMS2 proteins and the MSH2 and MSH6 proteins act as functional pairs, forming heterodimers [34]. Mutation of MLH1 or methylation of MLH1 typically results in loss of immunhistochemical expression of both MLH1 and PMS2. Similarly, mutation of MSH2 usually results in immunohistochemical loss of both MSH2 and MSH6. However, mutation of MSH6 alone usually is associated with loss of MSH6 protein but retention of MSH2 by immunohistochemistry. Similarly, mutation of PMS2 is typically associated with loss of PMS2 protein but retained MLH1 immunohistochemical expression.

### Which patients with endometrial cancer should be evaluated for Lynch syndrome?

Nationally, universal screening for LS in endometrial cancer patients is being advocated, and implemented by several centers. Algorithms for such screening have been developed [16,35]. In many centers, universal screening for CRC has already been implemented. The incidence in unselected populations of LS in EC patients is 2.3% [17], which approaches the 3% rate of LS in CRC patients. Usage of standard criteria such as the Amsterdam, Bethesda, or SGO criteria has led to underestimation of the true rate of EC in LS patients. Endometrial cancer is the most common gynecologic malignancy and is the sentinel event in LS 50% of the time.

Thus, we advocate for universal screening in endometrial cancer patients, with an age cut-off of 60, as the mean age of LS-related EC is over the age of 50. However, for EC patients who are older than 60, the screening test will be offered selectively based on clinicopathologic findings. This is mainly because that elderly patient may have a high tendency of MMR gene dysfunction. Patients should be screened first by immunohistochemical detection of the MMR proteins, not with MSI. If MLH1 loss is seen, hypermethylation of MLH1 should be performed. BRAF mutation does not have a major role in endometrial cancers, thus is not included in this screening algorithm. If there is a loss in MSH2, MSH 6, or PMS 2 alone, germline testing for mutations in those genes is recommended. If there is a loss in MLH1 not explained by hypermethylation, then germline MLH1 gene testing is recommended. If losses are seen in both MLH1 and PMS2, then the MLH1 loss is considered primary, and should be worked up as above. Similarly, if losses are seen in both MSH2 and MSH6, MSH2 is considered the primary event and germline testing for MSH2 is recommended (Figure 1, as an algorithm below).

**Figure 1:**
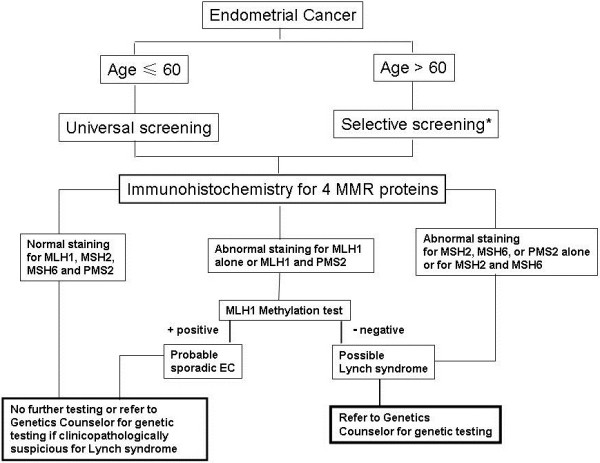
**Algorithm of screening patients with endometrial cancer for Lynch syndrome.** This algorithm does not cover the rare finding of Cowden’s Syndrome. Referral to the genetics counselor can always be made in any situation where the clinicopathologic suspicion is strong for Lynch syndrome.

In women older than 60 years, we suggest that cohorts be selected for screening. Both gynecologists and pathologists are in a position to identify EC patients who may be presenting with a sentinel manifestation of LS. Close interaction between gynecologists and gynecologic pathologists will further facilitate identifying LS in clinical practice. Others [15,36] have suggested selection criteria for LS screening of endometrial cancers. We advocate that the following clinical characteristics may provide important signs for clinicians that LS-related EC should be suspected with a diagnosis of EC:

(1) Patients, particularly with pathologic diagnosis as endometrioid carcinoma, show no evidence of estrogen overstimulation such as low body mass index (no obesity), no history of polycystic ovarian syndrome, nor unopposed estrogen usage;

(2) Patients with a synchronous endometrial and ovarian cancer, particularly clear cell carcinoma of the ovary;

(3) Patients with a family history of apparent LS;

(4) Patients with a personal history colon cancer;

Further, pathologists, particularly gynecologic pathologists, should be suspicious of LS for EC cases with the following pathologic characteristics:

(1) Endometrioid carcinoma without hyperplastic endometrium in the background;

(2) Heterogeneous cancer pathology such as one area with well differentiated carcinoma, but another area with different pathologic patterns such poor differentiation, mucinous features including signet ring cells, and medullary growth pattern;

(3) Tumor-infiltrating lymphocytes or a Crohn-like inflammatory infiltrate invading either front or periphery;

(4) Predominantly low uterine segment location after excluding cervical primary;

(5) Well differentiated (FIGO grade 1) endometrioid carcinoma but with more than 50% myometrial invasion;

(6) History of well differentiated endometrioid carcinoma with recurrence within 2 years after ruling out endometrial serous or clear cell carcinoma.

It is imperative that pathologists communicate with corresponding gynecologists prior to ordering the MMR protein immunohistochemical stains, if the institution is not prepared to implement universal screening for EC patients. Although the above clinicopathologic findings are neither specific nor sensitive, we think this may be a cost effective clinical method to identify patients at risk for LS prior to developing other LS related cancers. Based on the current understanding and clinical experience gained, we are advocating universal screening for patients who are 60 years or younger, and selective screening for those patients older than 60 years based on clinicopathologic findings (see screening algorithm above).

### Future directions

Our understanding of clinicopathologic features, pathogenesis, and the individual role of MMR gene mutation of LS-related ECs is continuing to evolve. Many studies including large scale clinical studies are needed to further characterize the above parameters in order to develop efficient surveillance systems and genetic tests in order to provide ideal diagnostic, therapeutic, and prevention modalities for the majority of LS-related cancers.
